# Molecular Phylogenetic Relationships, Trichothecene Chemotype Diversity and Aggressiveness of Strains in a Global Collection of *Fusarium graminearum* Species

**DOI:** 10.3390/toxins11050263

**Published:** 2019-05-11

**Authors:** Chami Amarasinghe, Barbara Sharanowski, W.G. Dilantha Fernando

**Affiliations:** 1 Department of Plant Science, University of Manitoba, 222 Agriculture Building, 66 Dafoe Road, Winnipeg, MB R3T 2N2, Canada; chami008@gmail.com; 2 Department of Biology, University of Central Florida, BIO441 Biological Sciences, 4110 Libra Drive, Orlando, FL 32816, USA; Barbara.Sharanowski@ucf.edu

**Keywords:** Fusarium Head Blight, mycotoxins, 3-ADON, 15-ADON, NIV, FGSC, trichothecene, deoxynivalenol, nivalenol, phylogenetics, wheat, *F. graminearum s.s*, *F. asiaticum*, *F. meridionale*, *F. cortaderiae*, *F. boothii* and *F. austroamericanum*, *EF-1α* gene

## Abstract

Fusarium head blight (FHB), caused principally by the species belonging to the *Fusarium graminearum* species complex (FGSC), is an important disease in wheat, barley, and other small grain crops worldwide. Grain infected with species in the FGSC may be contaminated with trichothecene mycotoxins such as deoxynivalenol (DON) and nivalenol (NIV). In this study, we characterized the phylogenetic relationships, chemotype diversity, phenotypic characters, and aggressiveness of 150 strains in FGSC collected from eight different countries. Phylogenetic analysis based on portions of translation elongation factor 1-α (*EF-1α*) gene from 150 strains revealed six species in the FGSC, *F. graminearum s.s*, *F. asiaticum*, *F. meridionale*, *F. cortaderiae*, *F. boothii,* and *F. austroamericanum*. In this collection, 50% of the strains were 15-acetyldeoxynivalenol (15-ADON), 35% were 3-acetyldeoxynivalenol (3-ADON) and 15% were NIV. Evaluation of strains on moderately resistant (MR) wheat cultivar Carberry indicated that there is no significant difference among the species for FHB disease severity (DS), fusarium damaged kernel percentage (FDK%) and DON production. However, significant differences were observed among the chemotypes. Results showed significantly higher FHB DS, FDK%, DON production, growth rates, and macroconidia production for the 3-ADON strains than the 15-ADON and NIV strains. In addition, significant differences for FHB response variables were observed among the strains from different countries. Our results demonstrate that type and amount of trichothecene produced by a strain play a key role in determining the level of aggressiveness of that particular strain, regardless of the species.

## 1. Introduction

FHB is economically one of the most important diseases of wheat throughout the world. Epidemics of FHB over the past 15 years in North America have had a devastating economic impact on agriculture [1,2]. Many FHB outbreaks have been reported in Canada, Asia, Europe, Australia, and South America suggesting that it is a major threat to world grain production [3,4]. One of the major concerns attributed with FHB is the contamination of grains with trichothecene mycotoxins and other estrogenic compounds. The *F. graminearum* species are capable of producing various B-trichothecenes, particularly, DON, its acetylated derivatives, 3-ADON and 15-ADON and NIV and its acetylated derivative, 4-acetyl nivalenol (4-ANIV) [5]. Based on the trichothecene profile, *F. graminearum* strains can be categorized into three main chemical groups or chemotypes namely, 3-ADON, 15-ADON, and NIV. A 3-ADON chemotype produces DON and 3-ADON, while a 15-ADON chemotype produces DON and 15-ADON and a NIV chemotype produces NIV and 4-ANIV [6,7,8]. In North America, DON is the primary mycotoxin in *Fusarium* infected grain, while in parts of Asia and Europe both DON and NIV are the common contaminants of grain [9]. These mycotoxins pose a significant threat to human and animal health [10]. Trichothecenes are also acutely phytotoxic and act as virulence factors on sensitive host plants [5].

Fusarium head blight is caused by several *Fusarium* spp. such as *F. graminearum*, *F. sporotrichioides*, *F. culmorum*, *F. cerealis*, *F. avenaceum*, *F. equiseti,* and *F. poae* [2]. Among these species of *Fusarium*, members of the FGSC are still considered to be the major etiological agents of FHB worldwide [9,11]. Other *Fusarium* species play a minor role in FHB development. Species of *Fusarium* have traditionally been classified based on the morphological characteristics such as the shape and size of the macroconidia, presence/absence of chlamydospores, and presence/absence, shape, and supporting structures of the macroconidia and microconidia [12].

Until the year 2000, members of the FGSC were considered a single cosmopolitan species as morphological approaches to species identification failed to accurately delimit species for this group. However, with the advances in DNA sequencing technology, sequence similarity at one or more diagnostic loci has become an important tool in determining the species limits with *Fusarium* spp. [12]. The sequences most widely used to identify species of *Fusarium* are portions of DNA sequences encoding *EF-1α*, β-tubulin, internally transcribed spacer (ITS) regions in the ribosomal DNA repeat region (ITS1 and ITS2), histone H3, and trichothecene biosynthesis genes, especially *TRI101* [8,11]. O’Donnell et al. [11] first identified seven phylogenetic species within the FGSC, using genealogical concordance phylogenetic species recognition (GCPSR). Later, using a high-throughput multilocus genotyping (MLGT) assay of portions of 13 housekeeping genes, coupled with GCPSR, another nine phylogenetically distinct, cryptic species were identified within the FGSC [13,14,15,16]. The species designation *F. graminearum* was therefore referred to as *sensu stricto* (*s. s.*) and retained for the species most commonly associated with FHB worldwide.

So far, 16 monophyletic species have been identified within the FGSC and these include: *F. austroamericanum* (lineage 1), *F. meridionale* (lineage 2), *F. boothii* (lineage 3) *F. mesoamericanum* (lineage 4), *F. acacia-mearnsii* (lineage 5), *F. asiaticum*, *F. graminearum sensu stricto* (lineage 7), *F. cortaderiae* (lineage 8), and eight additional monotypic lineages, including: *F. brasilicum*, *F. vorosii*, *F. gerlachii*, *F. aethiopicum*, *F. ussurianum*, *F. nepalense*, *F. louisianense,* and U.S. Gulf Coast population of *F. graminearum* [8,13,14,15,16,17]. Among the members of the FGSC, *F. graminearum s.s* has been found worldwide, while the distribution of other species was found to be restricted to certain geographic areas.

The *EF-1α* gene that encodes an essential part of the protein translation machinery has been extensively used to differentiate *Fusarium* spp. It has been shown that *EF-1α* provides much better resolution of relationships among and within lineages than other loci such as β-tubulin, calmodulin and ITS region [8,18]. Additionally, the absence of non-orthologous copies of *EF-1α* in the genus makes this locus a better candidate to resolve phylogenetic relationships among species. *EF-1α* gene has been used to investigate the species limits within the *F. graminearum* clade.

Recent studies have shown the emergence of new species within the FGSC and rapid population shifts that are characterized by changes in chemotype frequency [15,19]. The displacement of native 15-ADON chemotypes by a population with 3-ADON chemotypes in North America and emergence of new NIV-type and NX-2 populations in the USA have shown the importance of continued assessment of population shifts in FGSC [15,19,20,21]. Pathogenic and toxigenic differences between *F. graminearum* populations in combination with environmental factors are hypothesized to be the major reason for rapid shifts in pathogen populations. In recent years, many studies have been performed to examine species and trichothecene chemotypes diversity among FGSC strains [3,4,22,23]. Most of these studies were restricted either to a country or a particular region in a country. Very few studies have been undertaken to examine and compare the genetic diversity and pathogenicity among strains of FGSC associated with FHB from different countries [2,24,25]. Additionally, the findings from genetic diversity studies have been useful in making decisions in breeding for FHB resistant wheat cultivars. It has been reported that aggressiveness of *F. graminearum* strains collected from different regions within a country and even within populations from individual fields are highly variable, and therefore, FHB resistant wheat cultivars that are resistant in one region may not give consistent results in other regions [24,26]. The performance of a resistant cultivar mainly relies on the pathogen profile, environmental conditions and the interaction between these two variables in a specific location. Variation in pathogenicity and aggressiveness in pathogen populations can lead to host resistance being overcome [27,28]. Therefore, a better understanding of the pathogen profile, chemotype diversity and aggressiveness is important to align plant protection to the existing and potentially changing pathogen population, further; this may lead to improved strategies for disease management [27].

The members of the FGSC are highly variable in a number of morphological traits, including the size and shape of their conidia, growth rate on standard media, pathogenicity on wheat cultivars and the type of mycotoxins produced. Acquiring knowledge of the trichothecene mycotoxins produced by predominant FGSC populations in a cereal production region and further analysing their role in the pathogenesis are important to understand the factors affecting plant-pathogen interactions. Additionally, this will offer new approaches to breeding for FHB resistance. Therefore, the objectives of this study were to: (1) Determine the trichothecene chemotypes of *F. graminearum* strains based on *TRI3* and *TRI12* gene-specific polymerase chain reaction (PCR) assays; (2) evaluate the molecular phylogenetic relationships of FGSC strains collected from different countries based on the *EF-1α* gene; (3) determine the phenotypic characteristics of the strains in the FGSC; and (4) determine the aggressiveness of strains from different species in the FGSC.

## 2. Results

### 2.1. Identification of Fusarium Strains to Species Level

**Figure 1:**
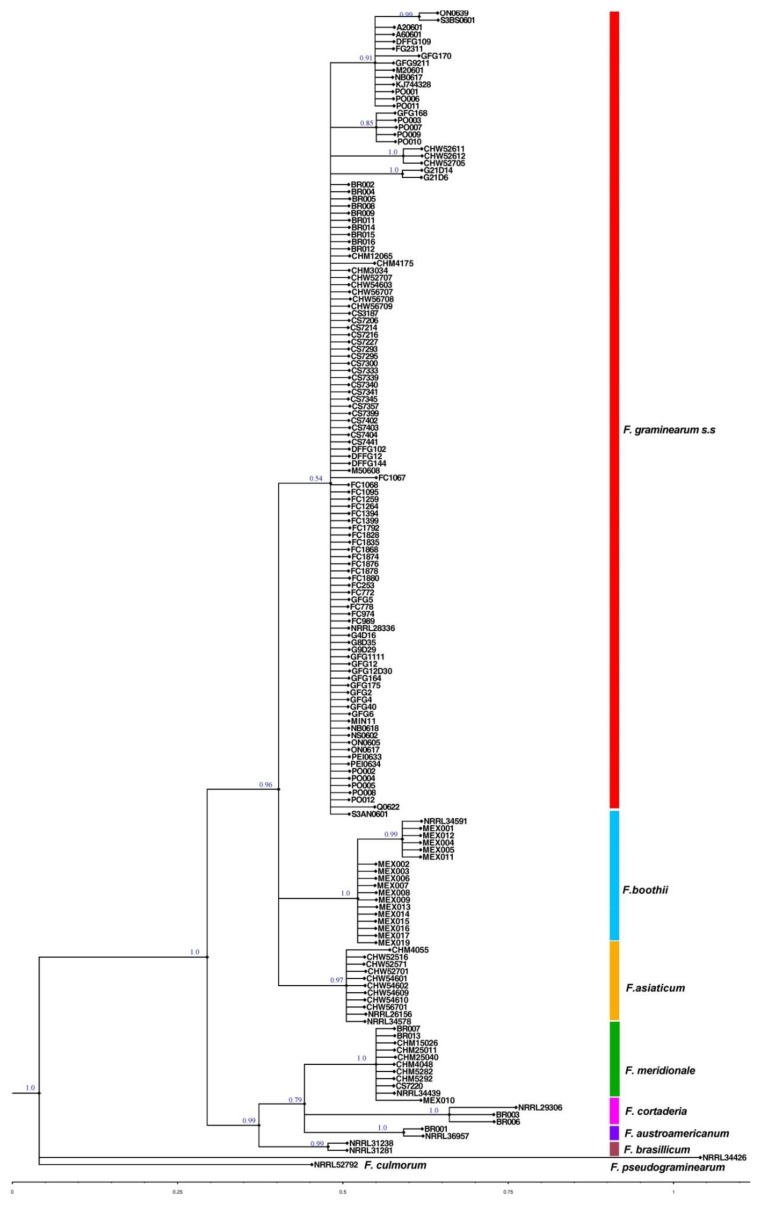
Inferred topology from the Bayesian analysis of *EF-1α*. Numbers at the nodes represent posterior probabilities.

Genetic polymorphisms in the DNA sequence of the *EF-1α* have been used to assist in the identification of 150 strains to species level. In the *EF-1α* analysis, 111 strains formed a monophyletic clade with reference strain *F. graminearum s.s* NRRL28336 confirming the strains identity as *F. graminearum s.s* (Figure 1). This clade includes strains from Australia, Poland, UK, Germany, Brazil, China and Canada. Another 17 strains from Mexico formed a monophyletic clade including the *F. boothii* reference strain NRRL34591. Within this clade, five strains from Mexico formed an intra-clade structure with *F. boothii* reference strain NRRL34591. Nine strains from China formed a monophyletic clade with *F. asiaticum* reference strains NRRL 26156 and NRRL 34578. Ten strains from the collection formed a monophyletic clade with *F. meridionale* reference strain NRRL 34439. This clade consists of strains originating from Australia, Brazil, China and Mexico. Two strains from Brazil formed a monophyletic clade with *F. cortaderiae* reference strain NRRL 29306. One strain from Brazil also formed a monophyletic clade with *F. austroamericarnum* reference strain NRRL 36957.

### 2.2. Trichothecene Chemotypes

The PCR assays based on *TRI3* and *TRI12* genes amplified products of 840, 610 and 243 bp corresponding with NIV, 15-ADON and 3-ADON chemotypes, respectively. Among the 111 *F. graminearum s.s* strains, 50% of the strains were of 15-ADON chemotype, 35% were 3-ADON producers and 15% were NIV producers (Supplementary Table S1). All NIV producing strains belonging to *F. graminearum s.s* were from Germany. Among the nine Chinese *F. asiaticum* strains, three strains were determined to be 3-ADON, three were NIV and another three were 15-ADON chemotypes. Among the 18 strains from Mexico, 17 strains were identified as *F. boothii* and all were 15-ADON producers. One strain was identified as *F. meridionale* and it was a NIV producer. All the *F. meridionale* strains in the collection (from Brazil, China and Australia) were identified as NIV producers. Also, the two strains of *F. cortaderiae* (from Brazil) were identified as an NIV chemotype. One strain from Brazil, which identified as *F. austroamericanum,* was determined to be a NIV chemotype.

### 2.3. Aggressiveness of Strains on Spring Wheat

The aggressiveness of 133 strains listed in Supplementary Table S1, except for strains obtained from Mexico, was evaluated on the MR spring wheat cultivar Carberry. Analysis of variance on FHB DS indicated that there were no significant differences among species. However, there were significant differences among the chemotypes. In addition, the two-way interaction between species and chemotypes was not significant (Table 1). *F. graminearum s.s* and *F. asiaticum* 3-ADON strains showed a comparatively higher DS than other species and chemotypes (Table 2). When we analysed the DS data according to the country of origin of strains, and the trichothecene chemotypes, DS was significantly different among the strains originating from different countries. The interaction country*chemotype was also significant. The 3-ADON chemotypes from Germany, UK, Poland, and China showed higher DS followed by Canadian 3-ADON strains (Table 2). We observed a similar pattern for FDK%, in which FDK% was not significantly different among the species, however, different among the chemotypes. The species*chemotype interaction was not significant. However, *F. graminearum* 3-ADON and *F. asiaticum* 3-ADON strains showed a higher FDK% compared to other species and chemotypes. In contrast to FHB DS, no significant difference was observed among the countries for FDK%. The two-way interaction, country*chemotype was also not significantly different. However, 3-ADON chemotypes from UK, Germany, China, Canada, and Poland showed higher FDK% than other chemotypes.

### 2.4. Deoxynivalenol/Nivalenol Content in Infected Grains

**Figure 2:**
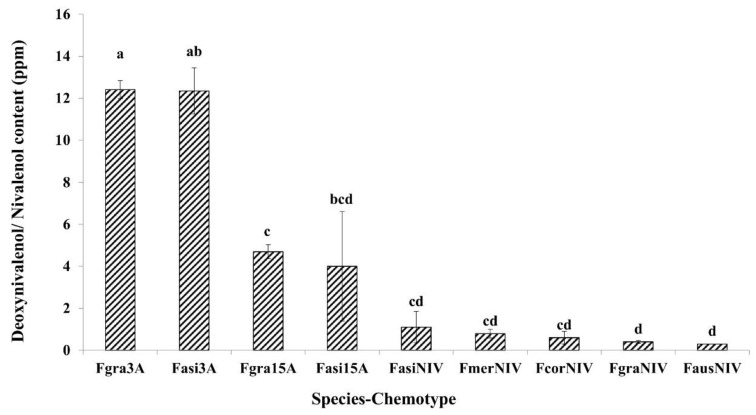
Comparison of mean deoxynivalenol/nivalenol production by chemotype of different species in the *Fusarium graminearum* species complex on moderately resistant wheat cultivar Carberry. Means with the same letter for deoxynivalenol/nivalenol content are not significantly different. (Fgra3A—*F. graminearum* 3-ADON chemotype; Fasi3A—*F. asiaticum* 3-ADON chemotype; Fgra15A—*F. graminearum* 15-ADON chemotype; Fasi15A—*F. asiaticum* 15-ADON chemotype; FasiNIV—*F. asiaticum* NIV chemotype; FmerNIV—*F. meridionale* NIV chemotype; FcorNIV—*F.* *cortaderiae* NIV chemotype; FgraNIV—*F. graminearum* NIV chemotype; and FausNIV—*F. austroamericanum* NIV chemotype). Bars on the top of the each column represent the standard errors.

The ANOVA for total DON/NIV content in infected grains showed no significant difference among species, however, there were significant differences among the chemotypes (Table 1). The interaction species*chemotype was also not significant. However, F. *gramineraum* 3-ADON and *F. asiaticum* 3-ADON strains produced higher amounts of DON in infected grains compared to other strains in the collection (Figure 2). In terms of DON/NIV content, significant differences were detected among countries and the country*chemotype interaction. The 3-ADON strains from Germany produced higher amounts of DON, followed by the 3-ADON strains from Canada, China, Poland, and UK. NIV producing strains showed the lowest toxin contamination in infected grains (Table 2).

**Figure 3:**
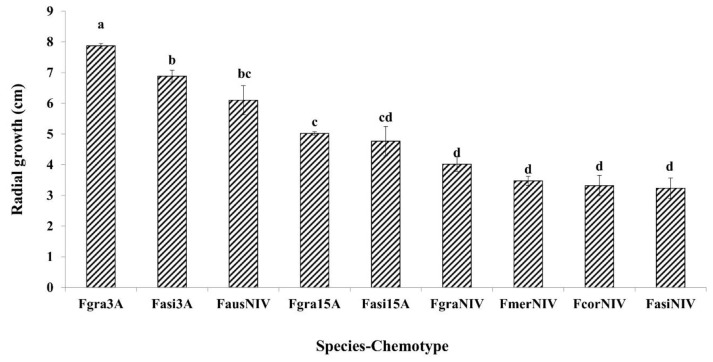
Comparison of mean radial growth by chemotype of different species in the *Fusarium graminearum* species complex on potato dextrose agar (PDA) media, seven days post-inoculation. Means with the same letter for mean radial growth are not significantly different. (Fgra3A—*F. graminearum* 3-ADON chemotype; Fasi3A—*F. asiaticum* 3-ADON chemotype; Fasi15A—*F. asiaticum* 15-ADON chemotype; Fgra15A—*F. graminearum* 15-ADON chemotype; FausNIV—*F. austroamericanum* NIV chemotype; FgraNIV—*F. graminearum* NIV chemotype; FmerNIV—*F. meridionale* NIV chemotype; FcorNIV—*F.* *cortaderiae* NIV chemotype; and FasiNIV—*F. asiaticum* NIV chemotype). Bars on the top of each column represent the standard errors.

**Figure 4:**
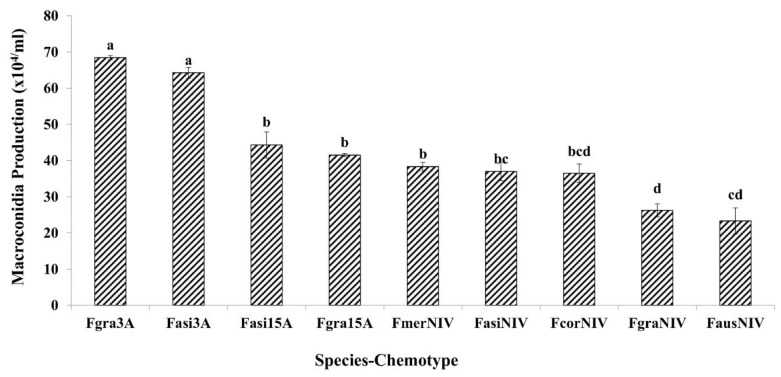
Comparison of mean macroconidia production by chemotype of different species in the *Fusarium graminearum* species complex on synthetic nutrient deficient agar (SNA) media, seven days post-inoculation. Means with the same letter for macroconidia production are not significantly different. (Fgra3A—*F. graminearum* 3-ADON chemotype; Fasi3A—*F. asiaticum* 3-ADON chemotype; Fasi15A—*F. asiaticum* 15-ADON chemotype; Fgra15A—*F. graminearum* 15-ADON chemotype; FmerNIV—*F. meridionale* NIV chemotype; FasiNIV—*F. asiaticum* NIV chemotype; and FcorNIV—*F.* *cortaderiae* NIV chemotype, FgraNIV—*F. graminearum* NIV chemotype; FausNIV—*F. austroamericanum* NIV chemotype). Bars on the top of each column represent the standard errors.

In the present study, significant differences were observed among the species and chemotypes in the FGSC for growth rate (Table 1). The two-way interaction species*chemotype was not significant. *F. graminearum s.s* 3-ADON, *F. asiaticum* 3-ADON and *F. graminearum* 15-ADON showed significantly higher growth rates than other species (Figure 3). Additionally, significant differences in growth rates were observed among the strains originated from different countries and the country*chemotype interaction was statistically significant (Table 2). The 3-ADON producing strains from Germany, UK, Canada, Poland, and China showed higher growth rates than the other chemotypes from the same countries. Similar results were observed for macroconidia production, except the two-way interaction species*chemotype was significantly different. *F. graminearum s.s* 3-ADON strains showed the highest macroconidia production on SNA media under in vitro conditions followed by *F. asiaticum* 3-ADON and *F. graminearum* 15-ADON strains (Figure 4). Canadian 3-ADON strains produced higher amounts of macroconidia under in vitro conditions followed by 3-ADON strains from Germany, Poland, UK and China (Table 2).

### 2.5. Correlation of FHB Disease Variables

To study the association between FHB disease variables, data on 132 strains were included in the correlation analysis. A significant positive correlation was observed between the FHB disease variables (Table 3). A highly significant positive correlation existed between FDK and DON/NIV content (*R* = 0.84, *P* < 0.0001). A significant positive correlation was also identified between DS and FDK (*R* = 0.72, *P* < 0.0001). Among the examined FHB disease variables, the lowest correlation was observed between the DS and macroconidia production (*R* = 0.64, *P* < 0.0001).

## 3. Discussion

In this study we conducted phylogenetic, in vitro and greenhouse experiments to compare the strains representing prevailing populations of the FGSC collected from different countries in relation to their trichothecene chemotype, phenotypic traits, and aggressiveness parameters. This type of comparative assessments for different species of the FGSC has not been exhaustively assessed since the subdivision of species in the FGSC has been proposed by O’Donnell et al. [11]. To our knowledge this is the first study that used *Fusarium* strains from multiple countries to analyze the phylogenetic relationships, chemotype diversity, and aggressiveness. Therefore, this study helps to understand the population subdivision in FGSC based on differences in geographic location, chemotype diversity, and aggressiveness.

Based on the *EF-1α* gene sequences six species in the FGSC could be identified in our *Fusarium* strain collection. All the strains examined from Canada, UK, Germany and Poland, most of the strains from Australia and Brazil and a few strains from China belong to the *F. graminearum s.s* clade. This finding agrees with other reports that show the wide geographical distribution of *F. graminearum s.s* among the other FHB species [2,9,17]. The strains from China belong to three different species, *F. asiaticum*, *F. graminearum s.s,* and *F. meridionale*. In Southern China, *F. asiaticum* is (approximately 97%) responsible for the major component of the FHB complex on wheat grown. However, in North and Northeast China, *F. graminearum s.s* (approximately 76%) was the most predominant species [29,30]. This uneven distribution of *Fusarium* species in the FGSC is believed to depend on the different crop rotation practices and temperatures in those regions [30,31]. Zhang et al. [32] reported the occurrence of *F. meridionale* strains on maize in China for the first time. All the examined strains from Mexico belong to the *F. boothii* group except for one strain which belongs to *F. meridionale*. The presence of *F. boothii* in Mexico has been previously reported by Malihipour et al. [33]. Similarly, all strains from Australia belong to the *F. graminearum s.s* except for one strain that was identified as *F. meridionale* (strain CS7220, isolated from wheat). Therefore, the discovery of NIV producing *F. meridionale* from Australia represents a potential risk for the Australian wheat industry.

Strains from Brazil consist of *F. graminearum s.s*, *F. cortaderiae*, *F. austroamericanum* and *F. meridionale*. Del Ponte et al. [4] reported the co-occurrence of different species in the *F. graminearum* species complex in Brazil. They identified five species within FGSC in a multiyear survey of >200 wheat fields in Paraná (PR) and Rio Grande do Sul (RS) states. These five species include *F. gramineraum s.s*, *F. meridionale*, *F. asiaticum, F. cortderiae,* and *F. austroamericanum*.

All the strains in this collection from European countries such as Germany, UK and Poland were identified as *F. graminearum s.s*. For example, in the Netherlands and Denmark, *F. culmorum* used to be the major causative agent of FHB before the year 2000; however, there was a population shift from *F. culmorum* to *F. graminearum* in Europe [34,35]. The dominance of *F. graminearum s.s* in European countries has also been reported by Jennings et al. [36] and Talas et al. [37]. Among the different species in FGSC, all strains of *F. boothii* were determined to be 15-ADON producers. The PCR assays and chemical analysis done by Malihipour et al. [33] and Sampietro et al. [38] also reported that *F. boothii* strains are capable of producing DON and 15-ADON. All strains of *F. meridionale*, *F. cortaderiae,* and *F. austroamericanum* were determined to be NIV producers. The trichothecene chemotypes detected in our collection are consistent with other reports of *F. meridionale*, *F. cortaderiae*, *F. austroamericanum,* and *F. boothii* from other parts of the world including Europe, China, Brazil, and Mexico [18,22,39].

Although many reports have been published on aggressiveness of *Fusarium* species and *F. graminearum* strains collected from a specific field or country, only a few studies have analyzed and compared the aggressiveness in species within the FGSC [25,40]. In our study we have evaluated and compared the aggressiveness of strains representing five phylogenetic species of the FGSC collected from different geographical regions. We used the spring wheat cultivar Carberry which expresses moderate resistance to FHB and has marker alleles associated with *Fhb1* [41]. To date, most selections in FHB resistant wheat breeding programs worldwide are concentrated on the *Fhb1* [42]. *Fhb1* is a major FHB resistance gene that is essential to provide Type II resistance in wheat cultivars against *F. graminearum* [43]. Therefore, use of a cultivar having *Fhb1* is important to evaluate the stability of resistance under the influence of different species and chemotypes of *F. graminearum*. In our study, in terms of *F. graminearum* species origin, no significant differences were observed for FHB DS, FDK% and DON/NIV content. However, significant differences were observed for radial growth and macroconidia production. Goswami & Kistler [2] also reported a large variation among different strains in terms of aggressiveness and trichothecene production. This variation appeared to be strain-specific rather than species-specific characteristics. In terms of chemotype of strains, significant differences were observed for all analysed FHB response variables. When we analyzed the data based on the country of origin of strains, significant differences were observed for all FHB response variables except for FDK%. Malihipour et al. [33] also compared the aggressiveness of *F. graminearum* strains from Canada, Iran, and Mexico and found significant differences in aggressiveness of the strains from different geographical regions. In the current study, country*chemotype interaction was also significantly different for FHB DS, DON/NIV content, radial growth and macroconidia production. However, country*chemotype interaction was not significantly different for FDK%. The 3-ADON producing strains from Europe (Germany, UK, and Poland) showed highest FHB DS in cultivar Carberry compared to other strains. When the total DON content in infected grains was considered, *F. graminearum s.s* 3-ADON showed the highest DON accumulation followed by *F. asiaticum* 3-ADON, *F. graminearum s.s* 15-ADON and *F. asiaticum* 15-ADON producing strains. Additionally, the 3-ADON producing strains from Germany showed the highest DON content followed by Canadian, Chinese and Poland strains. Apart from FHB DS and DON production, 3-ADON strains showed higher levels of FDK%, radial growth and macroconidia production compared to the 15-ADON and NIV strains. In our study, both *F. graminearum s.s* and *F. asiaticum* species showed higher aggressiveness than the other species of the FGSC. Other species in the FGSC such as *F. meridionale*, *F. austroamericanum*, and *F. cortaderiae* showed lower aggressiveness towards the MR wheat cultivar, Carberry. One of the reasons may be the production of less phytotoxic NIV by these species.

A study done by Eudes et al. [44] compared the phytotoxicity of eight different trichothecenes and reported that NIV is less phytotoxic than DON. Taken together, the results obtained from this study showed that, 3-ADON producing strains had the highest aggressiveness followed by 15-ADON and NIV producing strains, respectively. Similar results have been reported by Puri & Zhong [45], Malihipour et al. [33], Spolti et al. [46], and Zhang et al. [30] in which they also explained the higher aggressiveness of 3-ADON producing strains over 15-ADON and NIV strains. The lowest aggressiveness of NIV producing strains have also been reported in other studies [2,47]. The higher aggressiveness of 3-ADON producing strains in terms of DON production, mycelial growth, and macroconidia production may provide a fitness advantage over other chemotypes, suggesting that selection was driving the rapid spread of 3-ADON over 15-ADON and NIV chemotypes. Therefore, the higher aggressiveness and possibility of production of higher DON by 3-ADON strains in wheat cultivars introgressed with *Fhb1*, is a concern due to the rapid increase in 3-ADON producing strains in Canada and North America [21,48,49].

A significant correlation was observed between the amount of DON/NIV produced by each strain and its level of aggressiveness on wheat in terms of DS, FDK%, growth, and macroconidia production. Therefore, this study suggests that the type and amount of trichothecenes produced by a strain functions as a major determinant of aggressiveness on wheat. Additionally, our results are in agreement with previous reports showing that trichothecenes have a major role in determining the aggressiveness of the pathogen [37,50]. In terms of species origin of strains, we did not find a clear association between the species of a strain and its aggressiveness. However, the aggressiveness of strains may be partially based on species and population-specific features [51]. Therefore, systematic testing of many more strains representing different species, populations, and toxin types within the FGSC is required to understand this relationship in more detail.

In this study, we characterized the phylogenetic relationships, chemotype patterns, and aggressiveness of *Fusarium* strains in the FGSC collected from different regions, and evaluated the association between their phylogenetic and/or chemotype patterns with aggressiveness. The existence of high variability in *Fusarium* strains may explain the reasons for different reactions of wheat genotypes in different locations. Therefore, the results of the current study also suggest that screening for FHB resistance may require the use of highly aggressive strains or a mixture of strains representative of the FGSC diversity in order to develop durable FHB resistant wheat cultivars. Gaining knowledge of the phylogenetic relationships and trichothecene chemotype diversity in FGSC populations from major cereal producing regions/countries are also important to assess the sustainability of currently used resistant cultivars and fungicides. Further, it helps to establish quarantine regulations to limit pathogen spread among cereal growing regions.

## 4. Materials and Methods

### 4.1. Fusarium Strain Collection

One hundred and fifty *Fusarium* strains obtained from eight countries were included in this study (Supplementary Table S1). Strains were collected to represent different species in the FGSC from different regions in the world. All strains are stored in the culture collection maintained at Department of Plant Science, University of Manitoba, Canada.

### 4.2. DNA Extraction

All strains were grown on potato dextrose agar (PDA) (Difco Laboratories, ON, Canada) plates at 24–25 °C, under fluorescent light for 7 days and genomic DNA was extracted from the freeze-dried aerial mycelium using a modified cetyltrimethyl ammonium bromide (CTAB) based protocol described by Lodhiet al. [52]. The lyophilized mycelium (~200 mg) was ground in 600 µL of TES buffer (100 mM Tris, 10 mM EDTA, 2% SDS). Then 140 µL of 5M NaCl and 10% CTAB were added and vortexed. The mixture was incubated at 65 °C for 20 min. Protein was removed by adding 600 µL of a mixture of phenol:chloroform:isoamylalchohol (25:24:1). The mixture was centrifuged at 10,000 rpm for 15 min. This step was repeated twice. DNA was precipitated with 80 µL of 5M NaCl and 1000 µL of 95% ethanol. The mixture was centrifuged at 13,000 rpm for 5 min. The DNA pellet was washed using 200 µL of 80% ethanol. After the pellet dried, it was dissolved in 100 µL of autoclaved distilled water. DNA was treated with RNase (0.75% vol/vol). DNA was quantified using the NanoDrop3300 (Thermo Fisher Scientific Inc., Waltham, MA, USA). DNA was diluted using sterilized distilled water for a final concentration of 50 ng/μL and stored at −20 °C until further used.

### 4.3. PCR Assay and DNA Sequencing

Sequence analysis was performed for PCR-amplified fragments of *EF-1α* gene that has been used previously to discriminate between species in the FGSC. The primers used to amplify gene fragments are listed in Supplementary Table S2. The PCR reactions were performed in a 25 μL volume containing 20 ng of template DNA, 2.0 mM MgCl_2_, 50 mM KCl, 10 mM Tris HCl (pH 8.0), 0.2 mM each dNTP (Invitrogen Life Technologies, Carlsbad, CA, USA), 0.4 μM each primer, and 0.75 units of Taq DNA polymerase (Invitrogen Life Technologies, CA, USA). The PCR amplification protocol consisted of an initial denaturation at 94 °C for 2 min, followed by 30 cycles of 30 s at 94 °C, 60 s at annealing temperature, 1 min at 68 °C, and a final extension of 68 °C for 10 min. The annealing temperatures for the PCR amplification was 52 °C. For sequence analysis, PCR amplified DNA fragments were purified with Exosap-IT (Affymetrix Inc., Santa Clara, CA, USA) PCR product clean up kit according to the manufacturer’s instructions. Sequencing reactions were prepared using the BigDye Terminator v3.1 Cycle Sequencing Kit (Applied Biosystems, Foster City, CA, USA). Finally, the sequencing products were analyzed on an Applied Biosystems 3730xl DNA Analyzer (Applied Biosystems, CA, USA) at the University of Kentucky Advanced Genetic Technologies Center.

### 4.4. Molecular Phylogenetic Analysis

*EF-1α* gene sequences were assembled, trimmed and edited using GENEIOUS v. 5.4.5 [53]. Sequences were aligned manually using BIOEDIT v. 7.1.3 sequence alignment editor [54]. The final data set had an aligned length of 679 bp for *EF-1α* gene. Genetic distances and a test for base composition heterogeneity were performed using PAUP* v. 4.0 b10 [55]. A chi-square test of homogeneity of base frequencies across taxa was used to estimate the frequency distribution of observed number of substitutional changes per character. Base composition of aligned sequences was calculated in MEGA 5.1 beta version [56]. The Kimura 2-parameter plus Gamma (K80+G) and symmetrical (SYM) models were determined as the best fitting models of nucleotide substitution using the hierarchical likelihood ratio tests (hLRT) for *EF-1α* gene [57]. The model selection test was carried out using MODELTEST v. 3.7 [58]. Bayesian phylogenetic inference was performed using MrBayes v. 3.1.2 [59] with two independent runs with four chains each with default priors and run for 10^7^generations. Chains were sampled every 1000^th^ generation. Convergence of all parameters and correct mixing of chains were confirmed by examining the likelihood of plots for each run and when the average standard deviation of split frequencies was less than 0.02 [60]. Trees were summarized from the two independent searches using majority rule consensus after discarding 25% of the samples for burn-in. Calculation of the consensus tree and of the posterior probabilities of clades were done based upon the trees sampled after the burn-in, and trees were compiled and drawn using FIGTREE v. 1.3.1 [61]. *F. pseudograminearum* strain NRRL 34426 and *F. culmorum* strain NRRL 52792 were used as out-groups for *EF-1α* gene phylogenetic tree reconstruction. Additionally, sequences from Agricultural Research Services ARS (NRRL) culture collection reference strains were downloaded from GenBank and incorporated into each alignment.

### 4.5. PCR Assays to Determine Trichothecene Chemotypes

All primers used for the PCR-based identification of mycotoxin biosynthetic genes, along with references are presented in Supplementary Table S2. Chemotype identification of each strain was performed using multiplex PCR primers 3CON, 3NA, 3D3A, 3D15A described by Ward et al. [20]. The multiplex PCR primers amplified an 840 bp fragment from NIV producing strains, a 610 bp fragment from 15-ADON producers and a 243 bp fragment from 3-ADON producers, respectively. The PCR reactions were performed in 15 μL volumes containing 20 ng of template DNA, 2.0 mM MgCl_2_, 50 mM KCl, 10 mM Tris HCl (pH 8.0), 0.2 mM each dNTP (Invitrogen Life Technologies, Carlsbad, CA, USA), 0.4 μM each primer, and 0.75 units of Taq DNA polymerase (Invitrogen Life Technologies, CA, USA). The PCR cycling conditions for multiplex PCR consisted of an initial denaturation at 94 °C for 4 min, followed by 35 cycles of 1 min at 94 °C, 40 s at 52 °C, 40 s at 72 °C, and a final extension of 72 °C for 6 min. The PCR amplicons were separated on a 2% agarose gel stained with RedSafe nucleic acid staining solution (iNtRON Biotechnology Inc., FroggaBio, Toronto, ON, Canada).

### 4.6. Radial Growth and Macroconidia Production

The radial growth rate of each strain was evaluated in vitro on PDA plates in three replications. A small agar plug (5 mm in diameter) of each strain was placed in the center of a 9 cm PDA plate. The cultures were grown at 24 °C with a 24 h photoperiod. The average growth was measured every 48 h for six days from three replicates in two perpendicular directions. Average colony diameter was used to calculate the growth rate of the strain.

The macroconidia production was assessed based on the colonies growing on synthetic nutrient agar (SNA) media during seven days at room temperature and a 24 h photoperiod. After seven days of growth, conidia were harvested by adding 1 mL of sterilized distilled water and scraping the surface of SNA media with a sterilized scalpel. Number of conidia was determined using a haemocytometer and the conidial concentration was expressed in conidia/mL.

### 4.7. Aggressiveness Experiments and Mycotoxin Analysis

One hundred and thirty three strains listed in Supplementary Table S1 were individually inoculated on FHB MRwheat cultivar Carberry (except for the strains obtained from Mexico). Wheat plants were grown in plastic pots containing Sunshine Mix (Sun Gro Horticulture Ltd., Agawam, MA, USA). The pots were arranged in a completely randomized design with five replicates (one plant per pot). Inoculations were done using the dual floret point inoculation method. For each wheat plant, 4–5 spikes were inoculated once individual spikes were close to 50% anthesis. The point of inoculation on each spike was determined by calculating the total spikelets per spike and multiplied by two-thirds. Each spike was inoculated by injecting 10 µl of a macroconidial suspension (5 × 10^4^ spores/mL) between the lemma and palea of a floret. To facilitate infection, high humidity (>80%) was maintained around the inoculated spikes by placing a glassine bag over the spike. The bag was removed 48 h post-inoculation. FHB DSwas rated 14 days post-inoculation using a pictorial scale reported by Stack and McMullen [62].

After maturity, inoculated spikes were hand harvested and threshed using a belt thresher. A 10 g random seed sample was selected from pooled replicates and FDK% was counted as the number of FDKs in the total number of seeds. A FDK was considered to be any seed that was shrivelled, had any mycelial growth, or a chalky white or pink discoloration.

DON and NIV analysis was carried out using the same sample used for FDK determination. The samples were ground to a powder of similar consistency using a coffee grinder (Cuisinart model no: DCG20BKNC) for 5–8 min. DON was extracted using 50 mL of deionized water, and then quantified using Veratox DON 5/5 kit (product no: 8331) supplied by Neogen (Lansing, MI, USA) with a DON quantification limit (LOQ) of 0.1 ppm. The amount of NIV (LOQ-0.06 ppm) in infected grains was analyzed by GC-MS according to the protocol described by Amarasinghe et al. [49].

### 4.8. Statistical Analysis

Analysis of variance (ANOVA) for FHB DS, FDK%, DON/NIV content, radial growth, and macroconidia production was performed using ‘PROC MIXED’ procedure of the SAS software (SAS version 9.3, SAS Institute Inc., Cary, NC, USA). Because this data set has an unequal distribution of sample sizes, it was not possible to analyze the data set including all effects and their interactions. Therefore, to determine the effect of species and chemotype on FHB disease variables, data were analyzed using the following model statement; species country chemotype rep species*chemotype. A second data analysis was performed to determine the effect of country of origin and chemotype on FHB disease variables. The model statement for this analysis consisted of species country chemotype rep country*chemotype. The species, country, chemotype and their interactions (species*chemotype and country*chemotype) were considered as fixed effects and replicated as a random effect. The correlation between FHB response variables were analyzed using the SAS PROC CORR (SAS version 9.3, SAS Institute Inc., Cary, NC, USA) procedure.

## Figures and Tables

**Table 1:** Analysis of variance (ANOVA) table for fusarium head blight disease severity percentage (DS%), fusarium damaged kernel percentage (FDK%), deoxynivalenol (DON) or nivalenol (NIV) content in parts per million, radial growth in centimeters and macroconidia production in *Fusarium graminearum* species complex strains collected from different countries.

Trait	Source	DF	MS	F Value	Pr >F
DS%	Species	4	108.82	0.89	0.4695
	Country	6	3358.81	27.47	<0.0001
	Chemotype	2	12,181.00	99.61	<0.0001
	Species*Chemotype	8	327.51	2.69	0.0687
	Residual	636	122.28		
FDK%	Species	4	11.90	1.18	0.3253
	Country	6	9.52	0.94	0.4694
	Chemotype	2	2481.38	245.05	<0.0001
	Species*Chemotype	8	3.19	0.32	0.7303
	Residual	116	10.13		
DON/NIV (ppm)	Species	4	37.08	0.34	0.8509
	Country	6	28.80	4.6	0.0003
	Chemotype	2	395.18	63.19	<0.0001
	Species*Chemotype	8	7.01	1.12	0.3292
	Residual	116	6.25		
Radial growth (cm)	Species	4	4.00	6.35	<0.0001
	Country	6	5.70	9.04	<0.0001
	Chemotype	2	98.63	156.36	<0.0001
	Species*Chemotype	8	0.36	0.57	0.5670
	Residual	376	0.63		
Macroconidia	Species	4	294.52	7.94	<0.0001
	Country	2	270.51	7.29	<0.0001
	Chemotype	6	8772.54	236.57	<0.0001
	Species*Chemotype	8	393.36	10.61	0.0077
	Residual	376	37.08		

DF—Degree of Freedom; MS—Mean Square; Pr—Probability.

**Table 2:** Country of origin of strains, trichothecene chemotypes, mean values for fusarium head blight disease severity (DS%), fusarium damaged kernel percentage (FDK%), deoxynivalenol (DON) or nivalenol (NIV) content in parts per million, radial growth in centimeters, and macroconidia production of strains used in the study.

Country of Origin of Strains	Chemotype	DS%	FDK%	DON/NIV Content (ppm)	Radial Growth (cm)	Macroconidia (×10^4^/mL)
Germany	3-ADON	53.2 ^a^	38.0 ^a^	17.3 ^a^	8.5 ^a^	69.1 ^ab^
UK	3-ADON	48.1 ^ab^	35.7 ^a^	10.3 ^b^	8.1 ^ab^	66.2 ^b^
Poland	3-ADON	46.9 ^abc^	35.6 ^a^	11.0 ^b^	7.2 ^c^	67.7 ^ab^
China	3-ADON	44.8 ^abc^	36.0 ^a^	12.3 ^b^	6.8 ^c^	64.2 ^b^
Canada	3-ADON	42.1 ^bcd^	35.5 ^a^	12.6 ^b^	7.6 ^bc^	70.9 ^a^
China	15-ADON	39.1 ^cde^	14.1 ^b^	4.9 ^cd^	4.6 ^fg^	38.4 ^def^
Germany	15-ADON	35.8 ^de^	13.2 ^b^	5.7 ^c^	4.8 ^efg^	43.0 ^cd^
Canada	15-ADON	32.7 ^e^	15.7 ^b^	4.6 ^cd^	5.4 ^de^	43.6 ^cd^
UK	15-ADON	23.5 ^fg^	13.3 ^b^	5.2 ^cd^	5.0 ^def^	45.0 ^c^
Brazil	15-ADON	23.3 ^f^	16.1 ^b^	4.2 ^cd^	5.6 ^d^	38.4 ^def^
Poland	15-ADON	18.8 ^fgh^	13.8 ^b^	4.0 ^cd^	5.7 ^d^	33.6 ^efg^
Australia	15-ADON	16.6 ^gh^	13.6 ^b^	4.3 ^cd^	4.5 ^fg^	44.0 ^c^
Brazil	NIV	15.8 ^fgh^	5.8 ^c^	0.4 ^d^	4.1 ^gh^	32.8 ^fg^
Germany	NIV	14.3 ^fgh^	4.2 ^c^	0.3 ^d^	4.0 ^gh^	26.2 ^g^
China	NIV	13.0 ^h^	5.6 ^c^	1.1 ^d^	3.4 ^h^	40.0 ^cde^
Australia	NIV	9.8 ^fgh^	7.0 ^bc^	0.3 ^d^	2.8 ^h^	26.6 ^fg^

DS was recorded from wheat cultivar, Carberry and averaged from five replications, FDK and DON/NIV content were measured from a pooled sample obtained from five replications, radial growth and macroconidia production were obtained from three replications. Means with the same letter in the column for each variable are not significantly different.

**Table 3:** Pearson correlation coefficients of fusarium head blight response variables measured under controlled environment conditions and in the laboratory.

	FHB DS%	FDK % ^1^	Radial Growth	Macroconidia Production	DON/NIV Content ^2^
FHB DS%	**1.000**	0.72 * <0.0001	0.67 * <0.0001	0.64 * <0.0001	0.66 * <0.0001
% FDK		**1.000**	0.84 * <0.0001	0.90 * <0.0001	0.84 * <0.0001
Radial growth			**1.000**	0.79 * <0.0001	0.75 * <0.0001
Macroconidia production				**1.000**	0.80 * <0.0001
DON/NIV content					**1.000**

^1^ Fusarium Damaged Kernel Percentage, ^2^ Deoxynivalenol or Nivalenol Content * Means correlation coefficient is significant at *p* < 0.0001.

## Supplementary Materials

The following are available online at https://www.mdpi.com/2072-6651/11/5/263/s1. Table S1: Strain code, species, geographic origin, host and trichothecene chemotype of *Fusarium graminearum* species complex strains used in the study; Table S2: List of primers used to identify the species and chemotypes in the *Fusarium graminearum* species complex strains.
