# Supplementary material for: Molecular Phylogenetic Relationships, Trichothecene Chemotype Diversity and Aggressiveness of Strains in a Global Collection of Fusarium graminearum Species
Source: Toxins (Basel). 2019 May 11;11(5):263. doi: 10.3390/toxins11050263 (PMC6563009; doi:10.3390/toxins11050263)
Supplement: Supplementary file 1 [file toxins-11-00263-s001.pdf]

Supplementary Materials

Table S1. Strain code, species, geographic origin, host and trichothecene chemotype of *Fusarium graminearum* species complex strains used in the study.

	Species	Geographic origin	Host	Trichothecene chemotype
**CS7402**	*F. graminearum s.s*	Australia	Wheat	15-ADON
**CS7300**	*F. graminearum s.s*	Australia	Wheat	15-ADON
**CS7399**	*F. graminearum s.s*	Australia	Wheat	15-ADON
**CS7403**	*F. graminearum s.s*	Australia	Wheat	15-ADON
**CS7293**	*F. graminearum s.s*	Australia	Wheat	15-ADON
**CS7295**	*F. graminearum s.s*	Australia	Wheat	15-ADON
**CS7333**	*F. graminearum s.s*	Australia	Wheat	15-ADON
**CS7340**	*F. graminearum s.s*	Australia	Wheat	15-ADON
**CS7206**	*F. graminearum s.s*	Australia	Wheat	15-ADON
**CS7404**	*F. graminearum s.s*	Australia	Wheat	15-ADON
**CS7227**	*F. graminearum s.s*	Australia	Wheat	15-ADON
**CS7214**	*F. graminearum s.s*	Australia	Wheat	15-ADON
**CS7216**	*F. graminearum s.s*	Australia	Wheat	15-ADON
**CS7345**	*F. graminearum s.s*	Australia	Wheat	15-ADON
**CS7341**	*F. graminearum s.s*	Australia	Wheat	15-ADON
**CS7357**	*F. graminearum s.s*	Australia	Wheat	15-ADON
**CS3187**	*F. graminearum s.s*	Australia	Wheat	15-ADON
**CS7339**	*F. graminearum s.s*	Australia	Wheat	15-ADON
**CS7441**	*F. graminearum s.s*	Australia	Wheat	15-ADON
**CS7220**	*F. meridionale*	Australia	Wheat	NIV
**BR002**	*F. graminearum s.s*	Brazil	Wheat	15-ADON
**BR004**	*F. graminearum s.s*	Brazil	Wheat	15-ADON
**BR005**	*F. graminearum s.s*	Brazil	Wheat	15-ADON
**BR008**	*F. graminearum s.s*	Brazil	Wheat	15-ADON
**BR009**	*F. graminearum s.s*	Brazil	Wheat	15-ADON
**BR011**	*F. graminearum s.s*	Brazil	Wheat	15-ADON
**BR012**	*F. graminearum s.s*	Brazil	Wheat	15-ADON
**BR014**	*F. graminearum s.s*	Brazil	Wheat	15-ADON
**BR016**	*F. graminearum s.s*	Brazil	Wheat	15-ADON
**BR017**	*F. graminearum s.s*	Brazil	Wheat	15-ADON
**BR001**	*F.austroamericanum*	Brazil	Wheat	NIV
**BR003**	*F. cortaderiae*	Brazil	Wheat	NIV
**BR006**	*F. cortaderiae*	Brazil	Wheat	NIV
**BR007**	*F. meridionale*	Brazil	Wheat	NIV
**BR013**	*F. meridionale*	Brazil	Wheat	NIV
**NB0618**	*F. graminearum s.s*	Canada	Wheat	15-ADON
**Q0622**	*F. graminearum s.s*	Canada	Wheat	15-ADON
**DFFG109**	*F. graminearum s.s*	Canada	Wheat	15-ADON
**DFFG144**	*F. graminearum s.s*	Canada	Wheat	15-ADON
**DFFG102**	*F. graminearum s.s*	Canada	Wheat	15-ADON
**S3AN0601**	*F. graminearum s.s*	Canada	Wheat	15-ADON
**ON0617**	*F. graminearum s.s*	Canada	Wheat	15-ADON
**DFFG30**	*F. graminearum s.s*	Canada	Wheat	15-ADON
**ON0605**	*F. graminearum s.s*	Canada	Wheat	15-ADON

**PEI0634**	*F. graminearum s.s*	Canada	Wheat	15-ADON
**M50601**	*F. graminearum s.s*	Canada	Wheat	3-ADON
**M20601**	*F. graminearum s.s*	Canada	Wheat	3-ADON
**A20601**	*F. graminearum s.s*	Canada	Wheat	3-ADON
**NB0617**	*F. graminearum s.s*	Canada	Wheat	3-ADON
**A60601**	*F. graminearum s.s*	Canada	Wheat	3-ADON
**NS0602**	*F. graminearum s.s*	Canada	Wheat	3-ADON
**ON0639**	*F. graminearum s.s*	Canada	Wheat	3-ADON
**PEI0633**	*F. graminearum s.s*	Canada	Wheat	3-ADON
**MIN11**	*F. graminearum s.s*	Canada	Wheat	3-ADON
**NB0617**	*F. graminearum s.s*	Canada	Wheat	3-ADON
**S3BS0601**	*F. graminearum s.s*	Canada	Wheat	3-ADON
**DFFG120**	*F. graminearum s.s*	Canada	Wheat	3-ADON
**CHW52707**	*F. graminearum s.s*	China	Wheat	15-ADON
**CHW52611**	*F. graminearum s.s*	China	Wheat	15-ADON
**CHM4175**	*F. graminearum s.s*	China	Wheat	15-ADON
**CHM4055**	*F. graminearum s.s*	China	Wheat	15-ADON
**CHW52701**	*F. asiaticum*	China	Wheat	15-ADON
**CHW56708**	*F. graminearum s.s*	China	Wheat	15-ADON
**CHW56707**	*F. graminearum s.s*	China	Wheat	15-ADON
**CHM3034**	*F. graminearum s.s*	China	Maize	15-ADON
**CHM1265**	*F. graminearum s.s*	China	Maize	15-ADON
**CHW54601**	*F. asiaticum*	China	Wheat	3-ADON
**CHW54603**	*F. asiaticum*	China	Wheat	3-ADON
**CHW54611**	*F. asiaticum*	China	Wheat	15-ADON
**CH54604**	*F. asiaticum*	China	Wheat	NIV
**CHW54612**	*F. asiaticum*	China	Wheat	15-ADON
**CHW54602**	*F. asiaticum*	China	Wheat	3-ADON
**CHW52516**	*F. asiaticum*	China	Wheat	NIV
**CHWM25011**	*F. meridionale*	China	Maize	NIV
**CHM15026**	*F. meridionale*	China	Maize	NIV
**CHW52517**	*F. asiaticum*	China	Wheat	NIV
**CHM5282**	*F. meridionale*	China	Maize	NIV
**CHM5292**	*F. meridionale*	China	Maize	NIV
**CHM25040**	*F. meridionale*	China	Maize	NIV
**CHM4048**	*F. meridionale*	China	Maize	NIV
**GFG175**	*F. graminearum s.s*	Germany	Wheat	15-ADON
**G4D16**	*F. graminearum s.s*	Germany	Wheat	15-ADON
**GFG170**	*F. graminearum s.s*	Germany	Wheat	15-ADON
**GFG164**	*F. graminearum s.s*	Germany	Wheat	15-ADON
**GFG40**	*F. graminearum s.s*	Germany	Wheat	15-ADON
**GFG9211**	*F. graminearum s.s*	Germany	Wheat	15-ADON
**GFG2311**	*F. graminearum s.s*	Germany	Wheat	15-ADON
**GFG164**	*F. graminearum s.s*	Germany	Wheat	15-ADON
**GFG168**	*F. graminearum s.s*	Germany	Wheat	15-ADON
**G9D29**	*F. graminearum s.s*	Germany	Wheat	3-ADON
**GFG4**	*F. graminearum s.s*	Germany	Wheat	3-ADON
**GFG5**	*F. graminearum s.s*	Germany	Wheat	3-ADON
**GFG2**	*F. graminearum s.s*	Germany	Wheat	3-ADON
**G12D30**	*F. graminearum s.s*	Germany	Wheat	3-ADON
**GFG12**	*F. graminearum s.s*	Germany	Wheat	3-ADON

**G8D35**	*F. graminearum s.s*	Germany	Wheat	3-ADON
**G21D14**	*F. graminearum s.s*	Germany	Wheat	NIV
**G21D6**	*F. graminearum s.s*	Germany	Wheat	NIV
**GFG6**	*F. graminearum s.s*	Germany	Wheat	NIV
**GFG1111**	*F. graminearum s.s*	Germany	Wheat	NIV
**MEX001**	*F. boothii*	Mexico	Wheat	15-ADON
**MEX002**	*F. boothii*	Mexico	Wheat	15-ADON
**MEX003**	*F. boothii*	Mexico	Wheat	15-ADON
**MEX004**	*F. boothii*	Mexico	Wheat	15-ADON
**MEX005**	*F. boothii*	Mexico	Barley	15-ADON
**MEX006**	*F. boothii*	Mexico	Wheat	15-ADON
**MEX007**	*F. boothii*	Mexico	Wheat	15-ADON
**MEX008**	*F. boothii*	Mexico	Wheat	15-ADON
**MEX009**	*F. boothii*	Mexico	Wheat	15-ADON
**MEX011**	*F. boothii*	Mexico	Wheat	15-ADON
**MEX012**	*F. boothii*	Mexico	Wheat	15-ADON
**MEX013**	*F. boothii*	Mexico	Wheat	15-ADON
**MEX014**	*F. boothii*	Mexico	Wheat	15-ADON
**MEX015**	*F. boothii*	Mexico	Wheat	15-ADON
**MEX016**	*F. boothii*	Mexico	Wheat	15-ADON
**MEX017**	*F. boothii*	Mexico	Wheat	15-ADON
**MEX019**	*F. boothii*	Mexico	Wheat	15-ADON
**MEX010**	*F. meridionale*	Mexico	Wheat	NIV
**PO001**	*F. graminearum s.s*	Poland	Wheat	15-ADON
**PO002**	*F. graminearum s.s*	Poland	Wheat	15-ADON
**PO004**	*F. graminearum s.s*	Poland	Wheat	15-ADON
**PO005**	*F. graminearum s.s*	Poland	Wheat	15-ADON
**PO008**	*F. graminearum s.s*	Poland	Wheat	15-ADON
**PO003**	*F. graminearum s.s*	Poland	Wheat	3-ADON
**PO006**	*F. graminearum s.s*	Poland	Wheat	3-ADON
**PO007**	*F. graminearum s.s*	Poland	Wheat	3-ADON
**PO009**	*F. graminearum s.s*	Poland	Wheat	3-ADON
**PO010**	*F. graminearum s.s*	Poland	Wheat	3-ADON
**PO011**	*F. graminearum s.s*	Poland	Wheat	3-ADON
**PO012**	*F. graminearum s.s*	Poland	Wheat	3-ADON
**FC772**	*F. graminearum s.s*	UK	Wheat	15-ADON
**FC1878**	*F. graminearum s.s*	UK	Wheat	15-ADON
**FC1876**	*F. graminearum s.s*	UK	Wheat	15-ADON
**FC1874**	*F. graminearum s.s*	UK	Wheat	15-ADON
**FC1880**	*F. graminearum s.s*	UK	Wheat	15-ADON
**FC1835**	*F. graminearum s.s*	UK	Wheat	15-ADON
**FC778**	*F. graminearum s.s*	UK	Wheat	3-ADON
**FC1792**	*F. graminearum s.s*	UK	Wheat	3-ADON
**FC1068**	*F. graminearum s.s*	UK	Wheat	3-ADON
**FC253**	*F. graminearum s.s*	UK	Wheat	3-ADON
**FC1067**	*F. graminearum s.s*	UK	Wheat	3-ADON
**FC989**	*F. graminearum s.s*	UK	Wheat	3-ADON
**FC974**	*F. graminearum s.s*	UK	Wheat	3-ADON
**FC1868**	*F. graminearum s.s*	UK	Wheat	3-ADON
**FC1394**	*F. graminearum s.s*	UK	Wheat	3-ADON
**FC1095**	*F. graminearum s.s*	UK	Wheat	3-ADON

**FC1828**	*F. graminearum s.s*	UK	Wheat	3-ADON
**FC1264**	*F. graminearum s.s*	UK	Wheat	3-ADON
**FC1259**	*F. graminearum s.s*	UK	Wheat	3-ADON
**FC1399**	*F. graminearum s.s*	UK	Wheat	3-ADON

Table S2. List of primers and sequences used to identify the chemotypes of *Fusarium graminearum* species complex strains.

**Primer name**	**Gene**	**Sequence 5′-3′**	**Reference**
3CON	*TRI3*	TGGCAAAGACTGGTTCAC	Ward et al. [20}
3NA	*TRI3*	GTGCACAGAATATACGAGC	Ward et al. [20]
3D15A	*TRI3*	ACTGACCCAAGCTGCCATC	Ward et al. [20]
3D3A	*TRI3*	CGCATTGGCTAACACATG	Ward et al. [20]
EF-1	*EF-1α*	ATGGGTAAGGARGACAAGAC	O'Donnell et al. [8,11]
EF-2	*EF-1α*	GGARGTACCAGTSATCATGTT	O'Donnell et al. [8,11]
